# Circulating Biomarkers of Inflammation, Antioxidant Activity, and Platelet Activation Are Associated with Primary Combustion Aerosols in Subjects with Coronary Artery Disease

**DOI:** 10.1289/ehp.11189

**Published:** 2008-03-26

**Authors:** Ralph J. Delfino, Norbert Staimer, Thomas Tjoa, Andrea Polidori, Mohammad Arhami, Daniel L. Gillen, Micheal T. Kleinman, Nosratola D. Vaziri, John Longhurst, Frank Zaldivar, Constantinos Sioutas

**Affiliations:** 1 Department of Epidemiology, School of Medicine, University of California, Irvine, Irvine, California, USA; 2 Department of Civil and Environmental Engineering, Viterbi School of Engineering, University of Southern California, Los Angeles, California, USA; 3 Department of Statistics, School of Information and Computer Sciences; 4 Department of Community and Environmental Medicine, School of Medicine; 5 Department of Medicine, School of Medicine and; 6 Department of Pediatrics, School of Medicine, University of California, Irvine, Irvine, California USA

**Keywords:** acute-phase proteins, cytokines, enzymes, epidemiology, longitudinal data analysis, oxidative stress, panel study, particulate air pollution

## Abstract

**Background:**

Biomarkers of systemic inflammation have been associated with risk of cardiovascular morbidity and mortality.

**Objectives:**

We aimed to clarify associations of particulate matter (PM) air pollution with systemic inflammation using models based on size-fractionated PM mass and markers of primary and secondary aerosols.

**Methods:**

We followed a panel of 29 nonsmoking elderly subjects with a history of coronary artery disease (CAD) living in retirement communities in the Los Angeles, California, air basin. Blood plasma biomarkers were measured weekly over 12 weeks and included C-reactive protein (CRP), fibrinogen, tumor necrosis factor-α (TNF-α) and its soluble receptor-II (sTNF-RII), interleukin-6 (IL-6) and its soluble receptor (IL-6sR), fibrin D-dimer, soluble platelet selectin (sP-selectin), soluble vascular cell adhesion molecule-1 (sVCAM-1), intracellular adhesion molecule-1 (sICAM-1), and myeloperoxidase (MPO). To assess changes in antioxidant capacity, we assayed erythrocyte lysates for glutathione peroxidase-1 (GPx-1) and copper-zinc superoxide dismutase (Cu,Zn-SOD) activities. We measured indoor and outdoor home daily size-fractionated PM mass, and hourly pollutant gases, total particle number (PN), fine PM elemental carbon (EC) and organic carbon (OC), estimated secondary organic aerosol (SOA) and primary OC (OC_pri_) from total OC, and black carbon (BC). We analyzed data with mixed models controlling for temperature and excluding weeks with infections.

**Results:**

We found significant positive associations for CRP, IL-6, sTNF-RII, and sP-selectin with outdoor and/or indoor concentrations of quasi-ultrafine PM ≤ 0.25 μm in diameter, EC, OC_pri_, BC, PN, carbon monoxide, and nitrogen dioxide from the current-day and multiday averages. We found consistent positive but largely nonsignificant coefficients for TNF-α, sVCAM-1, and sICAM-1, but not fibrinogen, IL-6sR, or D-dimer. We found inverse associations for erythrocyte Cu,Zn-SOD with these pollutants and other PM size fractions (0.25–2.5 and 2.5–10 μm). Inverse associations of GPx-1 and MPO with pollutants were largely nonsignificant. Indoor associations were often stronger for estimated indoor EC, OC_pri_, and PN of outdoor origin than for uncharacterized indoor measurements. There was no evidence for positive associations with SOA.

**Conclusions:**

Results suggest that traffic emission sources of OC_pri_ and quasi-ultrafine particles lead to increased systemic inflammation and platelet activation and decreased antioxidant enzyme activity in elderly people with CAD.

Findings in cohort and time-series studies suggest that environmental exposure to particulate matter (PM) air pollution is associated with increases in cardiovascular hospitalization and mortality (Pope and Dockery 2006). Individuals at greatest risk have included elderly individuals with preexisting cardiovascular disease or other illnesses such as diabetes that place them at high risk for myocardial infarction or stroke.

Pathophysiologic mechanisms underlying the epidemiologic studies have been proposed and are under investigation (Utell et al. 2002). It is relevant in this regard that cohort studies have found that risk of cardiovascular events, including myocardial infarction, is associated with increased blood levels of inflammatory cytokines such as interleukin-6 (IL-6), tumor necrosis factor-α (TNF-α) and its receptors, adhesion molecules, and acute-phase proteins such as C-reactive protein (CRP) and fibrinogen (Pai et al. 2004). However, risk prediction based on these bio-markers may be in large part secondary to major modifiable environmental, behavioral, and clinical risk factors that are likely driving oxidative stress and inflammation (Folsom et al. 2006).

Among the potential environmental risk factors are air pollutants. It is possible, given the mounting toxicologic evidence, that air pollutant components may induce oxidative stress and inflammatory and prothrombotic responses by vascular endothelial cells, leukocytes, and platelets, with expression of inflammatory cytokines, cellular adhesion molecules, and coagulation factors (Mills et al. 2007). To clarify the relevance of these emerging experimental data, biomarker response data are needed in human subjects at increased cardiovascular risk.

In addition, the causal pollutant components driving the relationship of cardiovascular morbidity and mortality with PM remain to be identified. Historically, the difficulty in epidemiologic studies has been that the only available air pollution data originated from central regional ambient sites, which may not be representative of personal exposures, resulting in imprecise associations. Furthermore, the importance of particle size and chemistry has been limited by reliance on particle mass concentrations at two size cuts, PM_10_ (PM ≤ 10 μm in aerodynamic diameter) and PM_2.5_ (PM ≤ 2.5 μm). However, for the last several years, there has been mounting experimental evidence that ultrafine particles (UFPs; tentatively defined as PM < 0.1 μm in diameter) can induce the greatest amount of oxidative stress and inflammation per unit of PM mass (Cho et al. 2005; Ntziachristos et al. 2007). UFPs have high pulmonary deposition efficiency and particle number (PN) concentrations magnitudes higher than those for larger particles, and thus a much higher effective surface area. This large surface area carries high concentrations of toxic air pollutants such as polycyclic aromatic hydrocarbons (PAHs) and transition metals shown to induce oxidative stress responses (Li et al. 2003; Oberdörster 2001) and to translocate systemically from pulmonary sites (Elder and Oberdörster 2006). UFPs are a key size fraction emitted or formed close to sources of fossil fuel combustion that, as a result, leads to greater spatial variability than for larger size fractions. Therefore, exposure assessments of UFPs near the subject require special attention in epidemiologic research (Sioutas et al. 2005). Epidemiologic studies have shown that exposure markers for primary products of fossil fuel combustion point to important causal sources behind associations of particle mass with cardiovascular outcomes (Delfino et al. 2005).

We hypothesized that biomarkers of systemic inflammatory responses will be positively associated with ultrafine PM mass, total PN concentration, and markers of primary combustion-related organic compounds from sources such as vehicular exhaust. We further hypothesized that associations for predicted indoor exposure to PM of outdoor origin will be stronger than associations for unadjusted (raw) indoor exposures. To test these hypotheses, we conducted a study involving 12 repeated measurements of air pollutant exposures and circulating biomarkers of inflammation, antioxidant activity, and platelet activation in subjects with coronary artery disease (CAD) living in retirement communities within the Los Angeles, California, air basin. We collected air pollution data using a broad array of air monitors both in the immediate outdoor home environment and at indoor sites of the retirement communities studied. We took this approach to limit personal exposure error and allow detailed assessments of particle characteristics.

## Materials and Methods

### Design and population

In 2005–2006, we studied residents in independent living facilities of two retirement communities east of downtown Los Angeles. We started weekly follow-up with 33 subjects, but two subjects withdrew early and two subjects had too many infections (gastrointestinal and respiratory) to be included in the analysis, which excludes such observations. Over 7-month periods, we followed each subject with 12 weekly blood draws. We followed 18 and 13 subjects in the two retirement communities in four alternating 6-week phases. In each community, we collected 6 weeks of data during a warmer period of high photochemical activity (July to mid-October) and 6 weeks of data during a cooler period of higher air stagnation (mid-October to February).

We recruited subjects after referral to the study office by retirement-community nurses, or after self-referral through flyers and study presentations. Inclusion criteria included age ≥65; history of CAD diagnosis, which could include a history of myocardial infarction and bypass surgery but not within the preceding 12 months; and being sufficiently ambulatory to perform sit-to-stand transfers over short distances. Exclusion criteria included employment outside of the monitored home, smoking within the preceding 12 months, psychiatric disorders, dementia, alcohol or drug abuse, Parkinson or other debilitating neuromuscular diseases, dialysis treatment, daily oral corticosteroids, or medical conditions that would place the subject or staff at risk from the blood donation. The Institutional Review Board of the University of California, Irvine, approved the study protocol. We obtained informed written consent from all subjects.

### Blood collection and analysis

We drew venous peripheral blood samples at the same time of day and day of week to control for circadian rhythm and day-of-week effects. We used chilled anticoagulant Vacutainer tubes [ethylenediaminetetraacetic acid (EDTA) and citrate theophylline adenosine dipyridamole (CTAD) tubes]. Blood was rapidly separated (< 30 min after blood draw) into erythrocytes and plasma by using an onsite mobile field laboratory to minimize *ex vivo* changes in biomarkers. After centrifugation, each fraction was aliquoted, coded, transported frozen on dry ice from the field to our laboratory, and stored at –80°C until tested.

We measured concentrations of the following biomarkers in EDTA plasma using 96-well immunoassay kits: acute-phase proteins (fibrinogen and high-sensitivity CRP; Zymutest; Hyphen BioMed, Neuvillesur-Oise, France), fibrin D-dimer (marker of coagulation activation and fibrinolysis; Zymutest; Hyphen BioMed), proinflammatory cytokines (IL-6 and TNF-α; Quantikine HS; R&D Systems, Minneapolis, MN), soluble cytokine receptors [IL-6 soluble receptor (IL-6sR) and TNF-α soluble receptor II (sTNF-RII); Quantikine; R&D Systems], and myeloperoxidase (MPO; ALPCO Diagnostics, Salem, NH). MPO is primarily an indicator of neutrophil activation and is involved in the oxidative modification of lipoproteins. We analyzed soluble vascular cell adhesion molecule-1 (sVCAM-1) and soluble intracellular adhesion molecule-1 (sICAM-1) in plasma from CTAD tubes using a Linco Research Multiplex System (Linco Research, St. Charles, MO). We quantified soluble platelet selectin (sP-selectin), a marker of platelet activation (Jurk and Kehrel 2005), in CTAD-plasma by ELISA (R&D Systems). To examine changes in antioxidant capacity, we assayed erythrocyte lysates spectrophotometrically for activities of glutathione peroxidase-1 (GPx-1) and copper-zinc superoxide dismutase (Cu,Zn-SOD) using commercial test kits (Cayman Chemical, Ann Arbor, MI). The units of GPx-1 and Cu,Zn-SOD activities were normalized to units per gram of hemoglobin (Hb) in each sample (cyanmethemoglobin method). All samples of the same individual were analyzed in duplicate to ensure reproducibility and on the same microtiter plate to circumvent interassay variation.

### Exposures

We made indoor and outdoor home measurements of air pollutants and weather variables in the immediate vicinity of each retirement community. We measured size-fractionated 24-hr mean mass concentrations (μg/m^3^) of quasi-UFPs, 0–0.25 μm in diameter (PM_0.25_); accumulation-mode particles, 0.25–2.5 μm in diameter (PM_0.25–2.5_); and coarse mode particles, 2.5–10 μm in diameter (PM_2.5–10_). Considering that the upper size cut points traditionally used to define ultrafine mode particles (0.10–0.18 μm) are somewhat lower than the present cut point, PM_0.25_ is referred to here as “quasiultrafine.” We collected daily Teflon filter samples of PM_0.25_, PM_0.25–2.5_, and PM_2.5–10_ over 5 days (before the blood draw on Friday afternoons) with a Sioutas Personal Cascade Impactor Sampler using a 9 L/min pump (SKC, Inc. Eighty Four, PA). We made all other air pollutant measurements hourly for 9 days before the blood draw.

Indoor and outdoor hourly particle measurements included total PN concentration (particles/cm^3^) using the Condensation Particle Counter (model 3785; TSI, Inc., Shoreview, MN), and PM_2.5_ organic carbon (OC) and PM_2.5_ elemental carbon (EC) using two OC/EC analyzers (model 3F; Sunset Laboratory Inc., Tigard, OR). Particulate OC and EC were measured in hourly cycles (sampling time, 45 min; analysis time, 15 min). We placed a multichannel parallel carbon-plate diffusion denuder upstream of the OC/EC instrument to remove most of the organic vapors in the sampled air. We used a modified National Institute for Occupational Safety and Health analysis protocol to evolve particulate OC and EC. Elsewhere, we give a detailed description of all quality control and quality assurance analyses performed with the OC/EC analyzer (Arhami et al. 2006; Polidori et al. 2007). As an alternate measure of carbonaceous aerosols components, we measured outdoor hourly black carbon (BC) with an aethalometer (Magee Scientific, Berkeley, CA). Using standard reference methods, we measured hourly home pollutant gases, including indoor and outdoor nitrogen dioxide and carbon monoxide, and outdoor ozone.

We estimated concentrations of two general types of OC. Primary OC (OC_pri_) is emitted directly as products of incomplete combustion (e.g., PAH) from sources such as vehicular exhaust and wood smoke. Secondary organic aerosol (SOA) is formed when volatile reactive organic gases and semivolatile species (primarily aromatics in Los Angeles, but also alkanes, and biogenic monoterpenes and olefins) are oxidized to form low-volatility products that condense to produce organic PM (Manchester-Neesvig et al. 2003; Robinson et al. 2007). The contributions of OC_pri_ and SOA to measured outdoor OC were estimated from collected OC and EC concentrations using EC as a tracer of primary combustion-generated OC (i.e., “EC tracer method”) (Cabada et al. 2004). As described in detail by Polidori et al. (2007), this method assumes that OC_pri_ and EC are emitted from the same combustion sources. Typically, estimated SOA values were generally higher during the afternoon hours of summertime photochemical smog episodes.

Because our aim was to assess the effects of outdoor air pollutants, the indoor pollutants of interest are those of outdoor origin. Therefore, we first estimated air exchange rates and infiltration factors (F_inf_) at each site. We used estimated F_inf_ and measured particle concentrations across all hours in a single-compartment mass balance model to assess the contributions of indoor and outdoor sources to measured indoor EC, OC_pri_, SOA, and PN (Polidori et al. 2007). Thus, we determined the average amount of outdoor PM that penetrated inside the studied homes (outdoor-generated PM) by this approach. We assume that indoor exposures to PM of outdoor origin are relevant to personal exposures given that people in the retirement communities spend most of their time indoors.

### Analysis

We analyzed relationships of circulating biomarker concentrations to air pollutant exposures using linear mixed-effects models (Verbeke and Molenberghs 2001). This allows each subject to serve as his or her control over time (12 weekly measurements) and controls for potential confounding from between-subject covariates that do not change over time. Because the within-individual repeated measures of outcomes were correlated, and similarly so for the phase of study and retirement community, we assumed a two-stage hierarchical model with random effects at the subject level, nested within phase and community. We fit an autoregressive-1 correlation model given the covariance structure observed from empirical variograms. We conducted residual diagnostics to assess deviations from assumed functional form, and the presence of influential data points and subject clusters. Log transformation of biomarkers that exhibited skewed distribution (CRP, TNF-α, and MPO) did not improve the fit of the models or qualitatively change our findings.

We tested models for the 24-hr average period preceding the blood draw (exposure lag 0) as well as cumulative exposures up to 9 days (or 5 days for particle mass) before the blood draw to assess more acute versus cumulative exposure–response relationships, respectively. Data for exposure variables had to include ≥ 75% of the relevant exposure period.

We investigated potential confounding by weather variables. Temperature minimally influenced effect estimates, and we retained it in final models (same averaging time as the air pollutant). We decided *a priori* to adjust for acute infectious illnesses by excluding weeks with such observations given their known marked influence on biomarkers of inflammation. Medication variables were relatively stable during the 12-week follow-up and of more interest as effect modifiers. These include use of 3-hydroxy-3-methylglutaryl coenzyme A (HMG CoA) reductase inhibitors (statins), and angiotensin-converting enzyme (ACE) inhibitors or angiotensin II receptor blockers (ARBs). Use of these medications may dampen pollutant effects from their known protective influences on oxidative stress and inflammation. We tested a binary (yes/no) indicator for use of these medications and assumed that product term interactions with a *p*-value < 0.1 suggested possible effect modification.

## Results

### Descriptive data

Descriptive characteristics of the 29 subjects are shown in Table 1. Descriptive data for the biomarker measurements are shown in Table 2. We obtained 331 person-days of blood samples for biomarker measurements without any report of infection in the previous week (30 samples), ranging from 12 to 16 samples per week over the 24 weeks of follow-up (12 weeks per community).

Spearman correlations between biomarkers were strongest for IL-6 with TNF-α, sTNF-RII, sICAM-1, CRP, and fibrinogen (*r* range, 0.34–0.58) (not shown). Smaller correlations were observed for IL-6 with sP-selectin and sVCAM-1 (*r* range, 0.19–0.22) and among sTNF-RII, sP-selectin, CRP, and fibrinogen (*r* range, 0.18–0.28, except CRP and fibrinogen, *r* = 0.43). The strongest correlations were between IL-6 and CRP (*r* = 0.58) and between sICAM-1 and sVCAM-1 (*r* = 0.48). Cu,Zn-SOD was inversely correlated with sP-selectin (*r* = –0.29) and IL-6sR (*r* = –0.27).

Descriptive statistics for exposures are shown in Table 3. We previously identified indoor source contributions to PN and EC from cooking in our analysis of indoor PM of outdoor origin (Polidori et al. 2007), and this may explain why PM_0.25_ was higher indoors than outdoors. The relatively high outdoor PM_0.25_ reflects freshly emitted aerosols from local traffic sources of PM pollution in the Los Angeles area. A correlation matrix for air pollutants is shown in Supplemental Material, Table 1 (Supplemental Material is available online at http://www.ehponline.org/members/2008/11189/suppl.pdf). Markers for products of primary combustion were strongly correlated with each other (EC, BC, OC_pri_, PM_0.25_, NO_2_, and CO), whereas PN concentrations were moderately correlated with these pollutants. Indoor versus outdoor PM_0.25_, and indoor versus outdoor PM_0.25–2.5_ were strongly correlated. O_3_ was inversely correlated with PN and most of the markers of primary combustion.

### Regression analyses

#### Overview

**Figure 1:**
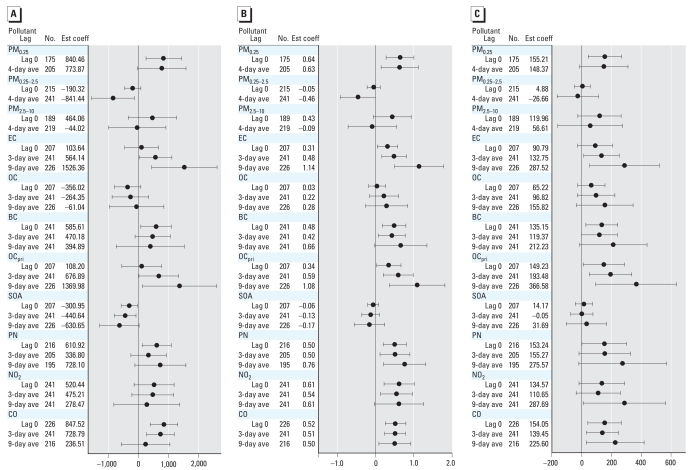
Relationships of biomarkers of inflammation to outdoor air pollutants (adjusted coefficient and 95% CI). (*A*) CRP (ng/mL). (*B*) IL-6 (pg/mL). (*C*) sTNF-RII (pg/mL). Expected change in the biomarker corresponds to an interquartile range change in the air pollutant (Table 3). Abbreviations: ave, average; Est coef, estimated coefficient.

**Figure 2:**
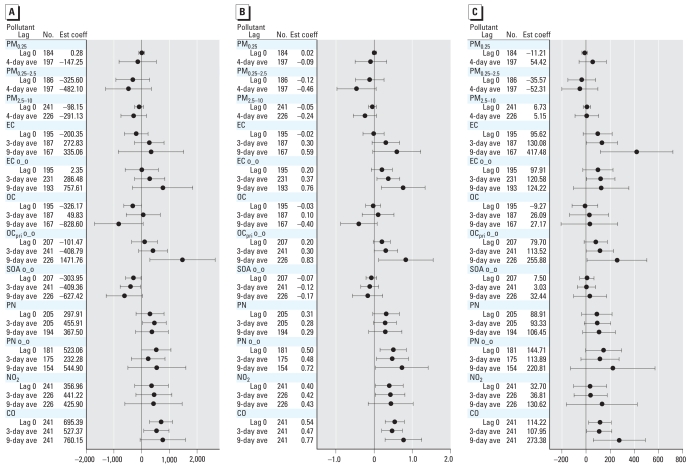
Relationships of biomarkers of inflammation to indoor air pollutants (adjusted coefficient and 95% CI). (*A*) CRP (ng/mL). (*B*) IL-6 (pg/mL). (*C*) sTNF-RII (pg/mL). Abbreviations: ave, average; Est coef, estimated coefficient; o_o, indoor concentrations of outdoor origin. Expected change in the biomarker corresponds to an interquartile range change in the air pollutant (Table 3).

**Figure 3:**
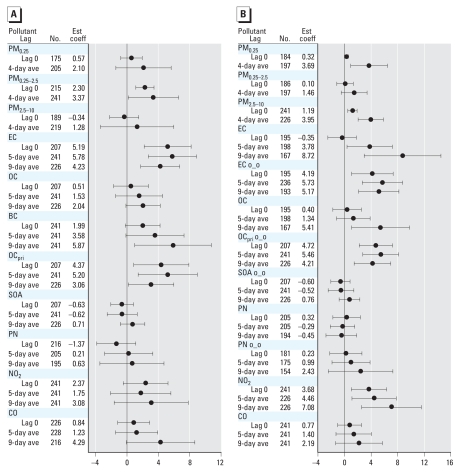
Relationship of sP-selectin to outdoor and indoor air pollutants (adjusted coefficient and 95% CI). (*A*) Outdoor air pollutants. (*B*) Indoor air pollutants. Abbreviations: ave, average; Est coef, estimated coefficient; o_o, indoor concentrations of outdoor origin. Expected change in sP-selectin (ng/mL) corresponds to an interquartile range change in the air pollutant (Table 3).

**Figure 4:**
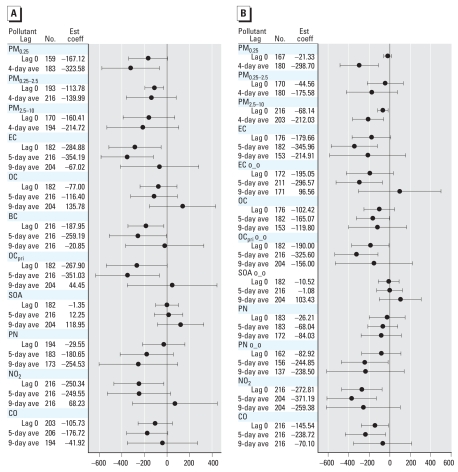
Relationship of Cu,Zn-SOD to outdoor and indoor air pollutants (adjusted coefficient and 95% CI). (*A*) Outdoor air pollutants. (*B*) Indoor air pollutants. o_o, indoor concentrations of outdoor origin; Est Coef, estimated coefficient. Expected change in Cu,Zn-SOD (U/g Hb) corresponds to an interquartile range change in the air pollutant (Table 3).

We found associations between several biomarkers and hourly air pollutants for lag 0 out to 9-day average concentrations. To simplify the presentation, we show models for lag 0 and for multiday averages for 3 days (or 5 days) and 9 days before the blood draw. We also found associations from lag 0 through the 4-day average of the various size-fractionated particle mass measurements. To simplify the presentation, we show models for lag 0 and 4-day averages. Overall, we observed significant (*p* < 0.05) associations for some but not all biomarkers and pollutant averaging times. The most significant positive associations with air pollutants were for CRP, IL-6, sTNF-RII, and sP-selectin (Figures 1–3). We also found significant inverse associations for Cu,Zn-SOD with air pollutants (Figure 4). Negative regression coefficients were also found for GPx-1 and MPO, but most were nonsignificant. Models for fibrinogen were not consistent with models for CRP and were mostly nonsignificant. Although most regression parameter estimates were positive, few were significant for TNF-α, sVCAM-1, or sICAM-1. Regression parameter estimates were largely negative and nonsignificant for the IL-6sR. Parameter estimates for fibrin D-dimer were close to zero for most models. Data tables for Figures 1–4 (including additional multiday average exposures) and for fibrinogen, TNF-α, sVCAM-1, sICAM-1, IL-6 receptor, GPx-1, and MPO are available in Supplemental Material, Tables 2–4 (Supplemental Material is available online at http://www.ehponline.org/members/2008/11189/suppl.pdf).

#### Biomarkers of inflammation (CRP, IL-6, and sTNF-RII)

Figure 1 shows the relationships of outdoor air pollutant exposures with these biomarkers. For size-fractionated particle mass data, outdoor quasi-ultrafine PM_0.25_ was positively associated with CRP, IL-6, and sTNF-RII. There was an unexpected inverse association of IL-6 and CRP with 4-day average outdoor accumulation-mode PM_0.25–2.5_. We observed positive associations of CRP, IL-6, and sTNF-RII with outdoor particulate and gaseous markers of primary combustion products (EC, BC, OC_pri_, NO_2_, CO) but not with SOA or total OC. Outdoor PN was positively associated with these biomarkers, as well. There was an unexpected inverse association of CRP with SOA.

Figure 2 shows the relationships of indoor air pollutant exposures with CRP, IL-6, and sTNF-RII. Unlike the outdoor data, none of the models for indoor particle mass was significant, and some parameters were negative. CRP and IL-6 were somewhat more strongly associated with indoor EC of outdoor origin than was uncharacterized indoor EC. CRP, IL-6, and sTNF-RII were also positively associated with indoor OC_pri_ of outdoor origin, but not total indoor OC or SOA of outdoor origin (some estimates were negative for CRP). We also observed stronger positive associations of IL-6 with indoor PN of outdoor origin than for uncharacterized indoor PN. Positive associations for indoor NO_2_ and CO were largely consistent with findings for outdoor gases.

#### Biomarker of platelet activation (sP-selectin)

Figure 3A shows the relationships of outdoor air pollutant exposures with this biomarker. Outdoor coarse PM_2.5–10_ was significantly associated with increased sP-selectin. Five-day average outdoor PM_0.25_ was nearly significant [2.95 ng/mL; 95% confidence interval (CI), –0.19 to 6.09; not shown]. We found significant positive associations of sP-selectin with most markers of primary combustion (EC, BC, OC_pri_) but not SOA. Only 9-day average total OC was nearly significant (*p* < 0.07).

Figure 3B shows the relationships of indoor air pollutant exposures with sP-selectin. Indoor PM_0.25_ and PM_2.5–10_ were positively and significantly associated with sP-selectin. We also observed stronger positive associations of sP-selectin with indoor EC of outdoor origin than for uncharacterized indoor EC. Indoor OC_pri_ of outdoor origin was positively associated with sP-selectin but not indoor SOA or PN of outdoor origin. Nine-day average total OC was significantly positive.

#### Biomarker of antioxidant activity (Cu,Zn-SOD)

Models for Cu,Zn-SOD were strongly influenced by three female subjects, resulting in a substantial decrease in the magnitude of regression parameter estimates. Influence statistics reflected this. For markers of primary combustion, these three subjects showed significant positive associations, whereas the remaining 26 showed significant inverse associations. For instance, for an interquartile increase in EC 5-day average, Cu,Zn-SOD increased by 2,678 U/g Hb in the three subjects (95% CI, 1,244 to 4,113), whereas in the remaining 27 it decreased by 571 U/g Hb (95% CI, –346, to –121). The only size fraction with significant positive associations in the three subjects was PM_0.25_, with similarly large increases of 2,794–4,881 U/g Hb for interquartile increases in all averaging times through the 5-day average (*p* < 0.005).

Figure 4A shows the relationships of outdoor air pollutant exposures with Cu,Zn-SOD, excluding the three subjects. All PM size fractions were inversely associated with Cu,Zn-SOD (outdoor PM_0.25_ and PM_0.25–2.5_ were statistically significant). We found inverse associations for outdoor particulate and gaseous markers of primary combustion products (EC, BC, OC_pri_, NO_2_, CO) but not SOA or total OC. Negative regression coefficients for PN were nonsignificant.

Figure 4B shows the relationships of indoor air pollutant exposures with Cu,Zn-SOD excluding the three subjects. Indoor particle mass associations were consistent with findings for outdoor pollutants. Associations were not stronger for indoor EC of outdoor origin compared with uncharacterized EC. However, associations were stronger for indoor PN of outdoor origin compared with uncharacterized PN. Cu,Zn-SOD was inversely associated with indoor OC_pri_ of out-door origin, whereas no associations were found for SOA of outdoor origin.

#### O_3_

We found inverse associations of O_3_ with CRP, IL-6, sTNF-RII, and sP-selectin [Supplemental Material, Table 2 (available online at http://www.ehponline.org/members/2008/11189/suppl.pdf)]. Conversely, O_3_ was positively associated with Cu,Zn-SOD in the 27 subjects. To address the possibility that the O_3_ association was spurious, we co-regressed O_3_ with EC for key biomarkers [Supplemental Material, Figure 1 (available online at http://www.ehponline.org/members/2008/11189/suppl.pdf)]. The associations with O_3_ apparent in single pollutant models were completely confounded by EC, whereas the associations with EC remained stable.

#### Effect modification by medication use

Biomarkers of inflammation showed differences in pollutant associations by statin use (*p* < 0.1). Among 13 subjects not on statins, CRP was more strongly associated with 3-day and 5-day averages of markers of primary combustion and with 7- and 9-day averages of PN. For example, among subjects not on statins, an interquartile increase in outdoor 3-day average EC was associated with an increase in CRP of 1,139 ng/mL (95% CI, 168 to 2,110), whereas there was no association among 16 subjects on statins (–45; 95% CI, –436 to 343) (interaction *p* < 0.03). There were also significantly stronger positive associations of CRP with outdoor PM_0.25_ among subjects not on statins. We found the same trend for IL-6 with PM_0.25_ and longer-term averages of markers of primary combustion (7- and 9-day averages). We found similar differences for sTNF-RII, PM mass (especially PM_0.25_), and markers of primary combustion, but with more averaging times from lag day 0 through 9-day averages [Supplemental Material, Figures 2 and 3 (available online at http://www.ehponline.org/members/2008/11189/suppl.pdf)].

An opposite pattern was found for sP-selectin. For example, among subjects on statins, an interquartile increase in outdoor 5-day average OC_pri_ was associated with an increase in sP-selectin of 13.4 pg/mL (95% CI, 6.2 to 20.6), whereas there was no association among subjects not on statins (–0.7; 95% CI, –3.2 to 1.8) (interaction *p* < 0.0005).

We also discovered significantly weaker associations of lag 0 through 5-day average EC and OC_pri_ with sP-selectin among 10 subjects taking the platelet aggregation inhibitor clopidogrel (3 also on coumadin) and among 16 subjects taking aspirin (4 also on clopidogrel). These medications did not confound pollutant interactions with statins in relation to sP-selectin. There was little to no evidence of differences in associations by ACE inhibitor or ARB use.

## Discussion

### Primary and secondary PM and particle size fraction

In a susceptible population of elderly individuals with CAD, we found positive associations of PM air pollution with biomarkers of systemic inflammation (CRP, IL-6, and sTNF-RII) and platelet activation (sP-selectin). These associations were observed primarily for markers of primary combustion sources (EC, BC, and OC_pri_) and were associated with exposures ranging from the current day to cumulative exposures 9 days in the past. We also observed inverse associations of enzymatic antioxidant activity (Cu,Zn-SOD) with PM for these exposures. We found some evidence for stronger biomarker associations for subjects not on statins. To our knowledge, the present analysis is among the first to estimate epidemiologic associations of cardiovascular outcomes with OC divided into two characteristics representing primary and secondary sources. This early finding is important because little is currently known about the pollutant components behind associations of cardiovascular hospitalization and mortality with ambient PM mass concentrations (Pope and Dockery 2006). Local primary combustion sources of air pollution are primarily from mobile sources in the Los Angeles area, and they generate high concentrations of redox-active organic components and metals (Cho et al. 2005; Ning et al. 2007; Ntziachristos et al. 2007).

Air pollutants from these sources readily penetrate into the indoor environment. This is the first study to our knowledge showing that associations of systemic biomarkers with outdoor PM are consistent with associations for indoor exposures to PM of outdoor origin, which in many (but not all) cases were stronger than the uncharacterized indoor exposures (EC, total OC, and PN). Results suggest that outdoor home measurements are sufficient to capture the cardiovascular health impacts of outdoor air pollutants even though people spend most of their time indoors. However, this does not apply to all outdoor pollutants. We found that “protective” effects of O_3_ were completely confounded by markers of primary combustion. This is attributable partly to O_3_ exposure error, given that little O_3_ remains stable indoors, leading to low indoor-to-outdoor ratios, especially with air conditioning use (Lee et al. 1999), which was frequent during hotter periods in our study communities.

We also found that PN (which is dominated by UFPs) and outdoor PM_0.25_ concentrations were more strongly and positively associated with biomarkers of inflammation (CRP, IL-6, sTNF-RII) than was PM_0.25–2.5_, supporting hypotheses regarding the pro-inflammatory potential of UFPs (Delfino et al. 2005). This may be attributable partly to the ability of UFPs to translocate systemically from pulmonary sites (Elder and Oberdörster 2006) and the potential of UFPs to promote oxidative stress (Cho et al. 2005; Li et al. 2003). When concentrations of PM_0.25_ and PM_0.25–2.5_ were combined to yield the typical fine particle mass metric of PM_2.5_, there was no evidence of associations with CRP, IL-6, or sTNF-RII (not shown). We also found significant associations of sTNF-RII, sP-selectin, and Cu,Zn-SOD with coarse particles (PM_2.5–10_). In the Los Angeles region, traffic contributes more to UFPs and coarse PM (due to brake abrasion and road dust resuspension) than it does to accumulation-mode particles (Sardar et al. 2005). This is consistent with the much stronger correlation of outdoor UFPs with coarse PM (*r* = 0.83) than with accumulation-mode PM (*r* = 0.44) [Supplemental Material, Table 1 (available online at http://www.ehponline.org/members/2008/11189/suppl.pdf)]. PM size fractions < 0.25 and > 2.5 μm are more short-lived in ambient air. Freshly generated reactive chemicals and transition metals on the particle surfaces of these two size fractions may explain the biologic responses we observed. Our SOA results are consistent with this hypothesis. SOA represent aerosols that are more aged and thus have a different chemical composition than do freshly generated aerosols. Because we do not have detailed chemical speciation for these particles (this is planned for the near future), we can only speculate on their relevance to effects.

We found evidence that PM_0.25–2.5_ was associated with increased sP-selectin and decreased Cu,Zn-SOD. However, inverse associations of PM_0.25–2.5_ and secondary OC with IL-6 and CRP are unexplained. They are not explained by interpollutant correlations between the markers of primary combustion and SOA or PM_0.25–2.5_, which were are all positive, albeit small. The lack of associations or weakened associations for indoor particle mass (especially for CRP, IL-6, and sTNF-RII) may be attributable to contributions from indoor sources such as cooking.

### Comparison with the literature

Our results for markers of primary combustion in the outdoor home environment point to the importance of traffic-related sources of pollutants. This is supported by a previous study showing that emergency department visits for acute myocardial infarction were associated with reports of in-vehicle exposures during the hours preceding symptom onset (Peters et al. 2004). Our results are also generally consistent with other recent longitudinal studies with repeated measures that have shown elevated ambient PM concentrations are associated with increased levels of circulating biomarkers of inflammation, although not always for the same sets of biomarkers (Chuang et al. 2007; Dubowsky et al. 2006; Rückerl et al. 2006, 2007a, 2007b; Yue et al. 2007). Our null finding for fibrin D-dimer is consistent with two other air pollution studies (Rückerl et al. 2006; Sullivan et al. 2007). In a panel study of subjects who had survived myocardial infarction, Rückerl et al. (2007a) found significant positive associations of IL-6 with central-site PN measurements, and fibrinogen with 5-day average PM_10_, but no significant associations with CRP. We found associations of PM_0.25_ with CRP but not fibrinogen. Ambient PM has been inconsistently associated with fibrinogen and CRP in various studies as discussed in Rückerl et al. (2007a).

Data are more scarce for sTNF-RII (Cox et al. 1999). sTNF-RII can compete with the membrane-bound forms of the TNF receptor and thus reduce TNF-dependent cell signaling (Heaney and Golde 1998). Significant positive associations with sTNF-RII and positive (but largely nonsignificant) coefficients for TNF-α point to an up-regulated inflammatory response to PM. This difference in significance for the receptor and cytokine may be attributable to the longer half-life and higher levels of the soluble receptor than of TNF-α in plasma. Additional data are needed to test the importance of sTNF-RII as a biomarker of the proinflammatory effects of air pollutants.

Our findings for sP-selectin suggest that air pollutants can induce platelet activation, which is supported by human experimental data (Törnqvist et al. 2007). P-selectin is critical for initial leukocyte adhesion to platelets and endothelial cells, and activates leukocytes and endothelial cells at sites of arterial injury (Jurk and Kehrel 2005). Clinical studies have shown that clopidogrel is associated with decreased sP-selectin (Xiao and Théroux 2004), which may explain our finding smaller magnitudes of association between air pollutants and sP-selectin in subjects taking clopidogrel. A similar panel study of 57 men with CAD showed central-site ultrafine PN concentrations in Erfurt, Germany, was positively associated with soluble CD40 ligand, another marker of platelet activation (Rückerl et al. 2007b).

### Antioxidant activity

A novel finding is the inverse associations of exposure to primary combustion aerosols with the antioxidant erythrocyte enzyme Cu,Zn-SOD (Figure 4) and, to a less significant extent, GPx-1 [Supplemental Material, Tables 2–4 (available online at http://www.ehponline.org/members/2008/11189/suppl.pdf)]. We initially hypothesized that antioxidant activity would increase in response to exposure (this was the case in three subjects). Diminished antioxidant response could lead to oxidative stress and then inflammation, with increased susceptibility to adverse cardiovascular events. The potential importance of this finding is shown in prospective cohort studies that found a low level of erythrocyte GPx-1 activity was associated with increased risk of nonfatal myocardial infarction or death from cardiovascular causes in patients with CAD (Espinola-Klein et al. 2007).

Erythrocytes account for much of the antioxidant capacity of the blood and are packed with enzymes that handle intracellular oxidative stress from the continuous endogenous generation of oxygen radicals (Tsantes et al. 2006). Pollutant-induced increases in oxidative or nitrosative stress throughout the circulation could overburden erythrocytic antioxidant defense. An *in vitro* study using pulmonary macrophages and erythrocytes showed that UFPs enter these cells by diffusion or adhesive interactions, and particles within cells were not membrane bound, thereby having direct access to intracellular proteins (Geiser et al. 2005). Other data show that PAHs can be transferred from the UFP surface to the cell membrane and then cross the membrane into the cytosol (Penn et al. 2005). UFPs and their redox cycling pollutant components could thus induce oxidative stress as shown with macrophage and epithelial cell cultures (Li et al. 2003).

Our finding of reduced activity of erythrocyte Cu,Zn-SOD and GPx-1 may be partly a consequence of enzyme inactivation by free radicals (Pigeolet et al. 1990). This could occur through oxidative modification of amino acids or irreversible modification of enzyme function by electrophiles (Shinyashiki et al. 2008). *In vitro* data using enzyme suspensions have shown marked reduction in the activity of GPx-1, Cu,Zn-SOD, and/or manganese SOD from variety of particle surrogates (Hatzis et al. 2006). GPx-1 and Cu,Zn-SOD activities decreased in the lungs of rats following intratracheal instillation of diesel exhaust particles (Sagai et al. 1993).

### Strengths and limitations

The primary strength of our findings is that we measured exposures in the microenvironments where subjects spend most of their time, and we designed the measurements to identify causal pollutant characteristics, which cannot be inferred from regulated particle mass (PM_2.5_ or PM_10_) from regional monitoring stations. The calculation of OC_pri_ and secondary OC combined with an estimation of indoor exposure to PM of outdoor origin provided among the first data showing associations between indoor urban particle components and systemic biomarkers in free-living humans. This addresses a critical weakness in the use of central regional data in that it does not capture the high spatial variability of pollutants from proximity to sources of primary combustion gases and aerosols, which include UFPs and their associated high concentrations of redox active components (Ntziachristos et al. 2007). Another year of data collection and analysis is planned (62 total subjects), and we anticipate that this will strengthen the present findings.

Our study also has several limitations. Although the data were collected in the home environment of subjects, we were not able to correct for exposures in other environments, which is usually accomplished with personal air monitors. In addition, we could perform only a limited assessment of differences in association based on clinical characteristics, but we anticipate sufficient power to do so with a planned doubling of sample size.

We tested multiple outcomes and exposures, potentially increasing the probability of a type 1 error. However, this was not an exploratory study without any prior knowledge or hypotheses; thus, corrections such as Bonferroni are inappropriate. Furthermore, the strong dependence among the air pollutants and among the biomarkers argues against correction of multiple testing by the number of tests (Bender and Lange 2001). We selected the biomarkers and exposure measurements to represent parts of key biologic pathways and pollutant characteristics, respectively, that were representative of our hypotheses. However, it is likely that the biomarkers are not all equivalent in their sensitivity to pollutant-induced responses and that the exposures are not all equivalent in their representation of causal pollutant components. In this sense, the exploratory nature of the research is to provide data for the selection of the best biomarkers (e.g., IL-6, sP-selectin) and exposures (e.g., EC or BC) for future research. The challenge will be to evaluate the impacts of air pollution on the inflammatory cascade of events that begins with oxidative stress and may end with thrombosis (two phenomena that we did not adequately characterize here).

## Conclusions

Our results suggest that traffic-related emission sources of PM_2.5_ OC_pri_, and UFPs lead to increases in systemic inflammation, platelet activation, and decreases in erythrocyte antioxidant activity in elderly people with a history of CAD. This is strongly supported by our findings of generally more robust associations for indoor PM of outdoor origin (PN concentration, PM_2.5_ OC_pri_, and EC), particularly given that people spend most of their time indoors. We found little to no evidence for associations with secondary PM_2.5_ OC or total OC, perhaps because the biologic responses we observed were triggered by freshly generated reactive chemicals on particle surfaces. UFPs are particularly enriched with redox-active chemicals. This is important because oxidative stress may be a mechanism whereby air pollutants induce inflammation in the airways and systemically, leading to acute adverse cardiorespiratory responses. The increase in oxidative (or nitrosative) stress can occur through pollutant-induced increases in reactive oxygen (or nitrogen) species combined with decreases in antioxidant enzyme activity (as we have shown). Diminished enzymatic antioxidant activity in erythrocytes could reflect increased plasma oxidative/nitrosative demands, which results in systemic inflammation as indicated in our study by CRP, sTNF-RII, IL-6, and sP-selectin. The public health relevance of our findings is that they come from the everyday life of a susceptible population living in a large U.S. city with infrequent violations of U.S. National Ambient Air Quality Standards during the study (South Coast Air Quality Management District 2007).

## Figures and Tables

**Table 1:** Characteristics of subjects.

Characteristic	Mean ± SD or no. (%)
Age (years)	85.7 ± 5.94
BMI (kg/m^2^)	26.6 ± 4.46
Sex	
Male	12 (41.4)
Female	17 (58.6)
Cardiovascular history	
Confirmation of CAD^a^	
Myocardial infarction	10 (34.5)
Coronary artery bypass graft or angioplasty	10 (34.5)
Positive angiogram or stress test	6 (20.7)
Clinical diagnosis	3 (10.3)
Current angina pectoris	9 (31.0)
Pacemaker or defibrillator	8 (27.6)
Cardiac arrhythmia	11 (37.9)
Congestive heart failure	10 (34.5)
Hypertension	22 (75.9)
Hypercholesterolemia	18 (62.0)
Other medical history	
Type II diabetes	5 (17.2)
COPD or asthma	4 (13.8)
Stroke or transient ischemic attack	5 (17.2)
Medications	
ACE inhibitors and ARBs	15 (51.7)
HMG CoA reductase inhibitors (statins)	16 (55.2)
Platelet aggregation inhibitors^b^	10 (34.5)
Aspirin	16 (55)
Calcium channel blockers	9 (31.0)
Smoking history	
Never smoker	25 (86.2)
Ex-smoker (no smoking preceding 12 months)	4 (13.8)

Abbreviations: BMI, body mass index; COPD, chronic obstructive pulmonary disease.

a Each category is hierarchical and excludes being in the above diagnostic category.

b Three were also taking coumadin.

**Table 2:** Biomarker concentrations (331 measurements).

Biomarker^a^	Mean ± SD	Median (Min–Max)
IL-6 (pg/mL)	2.95 ± 2.32	2.45 (0.28–20.14)
IL-6sR (ng/mL)	43.0 ± 13.9	40.8 (12.1–124)
TNF-α (pg/mL)^b^	1.82 ± 2	1.18 (0.5–14.3)
sTNF-RII (pg/mL)	3,933 ± 1,555	3,502 (658–11,584)
sP-selectin (ng/mL)	37.6 ± 15.1	36 (6–221)
sVCAM-1 (ng/mL)	787 ± 252	751 (203–1,639)
sICAM-1 (ng/mL)	155 ± 52	159 (74–383)
CRP (ng/mL)^c^	3,134 ± 3,796	1,938 (250–26,799)
Fibrinogen (μg/mL)	3,542 ± 1,200	3,299 (500–9,149)
D-dimer (ng/mL)^d^	820 ± 527	686 (250–3,366)
Cu,Zn-SOD (U/g Hb)	5,260 ± 1,671	5,018 (669–13,138)
GPx-1 (U/g Hb)	14.7 ± 5.6	14.2 (5.5–47)
MPO (ng/mL)	27.7 ± 30.7	17.6 (5–298)

Abbreviations: Max, maximum; Min, minimum.

a Excludes observations for weeks when there was a reported infection.

b Values of TNF-α < 0.5 could not be measured and were set to 0.5.

c Values of CRP < 250 could not be measured and were set to 250.

d Values of D-dimer < 250 could not be measured and were set to 250.

**Table 3:** Descriptive statistics of daily air pollutant measurements.

Exposure (24-hr averages)	No. (missing)	Mean ± SD	Median	IQR	Min–Max
Outdoor hourly PM					
EC (μg/m^3^)	166 (14)	1.61 ± 0.62	1.56	0.92	0.24–3.94
OC (μg/m^3^)	166 (14)	5.94 ± 2.11	5.58	2.79	2.51–13.60
BC (μg/m^3^)	179 (1)	2.00 ± 0.77	1.89	0.96	0.58–5.11
OC_pri_ (μg/m3)	166 (14)	3.37 ± 1.21	3.21	1.63	0.99–7.11
Secondary OC (μg/m^3^)	166 (14)	2.49 ± 1.50	2.10	1.86	0–8.10
PN (particles/cm^3^)	152 (28)	16,043 ± 5,886	13,968	7,386	6,837–31,263
Outdoor hourly gases					
NO_2_ (ppb)	178 (2)	33.10 ± 9.64	32.22	13.87	11.22–59.83
CO (ppm)	169 (11)	0.71 ± 0.29	0.70	0.41	0.14–1.68
O_3_ (ppb)	176 (4)	33.22 ± 11.68	32.19	15.69	8.50–70.88
Indoor hourly PM					
EC (μg/m^3^)	148 (32)	1.31 ± 0.52	1.30	0.70	0.19–2.89
EC of outdoor origin (μg/m^3^)	158 (22)	1.11 ± 0.39	1.06	0.51	0.41–2.97
OC (μg/m^3^)	148 (32)	5.69 ± 1.51	5.60	1.96	2.34–10.79
OC_pri_ of outdoor origin (μg/m^3^)	166 (14)	2.18 ± 0.82	2.15	1.07	0.32–5.21
Secondary OC of outdoor origin (μg/m^3^)	166 (14)	2.08 ± 1.26	1.75	1.45	0–6.87
PN (particles/cm^3^)	144 (36)	14,494 ± 6,770	12,341	7,337	1,016–43,027
PN of outdoor origin (particles/cm^3^)	124 (56)	10,108 ± 3,108	9,580	3,684	1,016–17,700
Indoor hourly gases					
NO_2_ (ppb)	170 (10)	32.3 ± 8.5	32.0	11.0	12.5–53.5
CO (ppb)	174 (6)	0.78 ± 0.30	0.73	0.42	0.22–1.97
Outdoor PM mass					
PM_0.25_ (μg/m^3^)	106 (14)	9.47 ± 2.97	9.4	4.2	3.31–18.75
PM_0.25–2.5_ (μg/m^3^)	115 (5)	13.53 ± 10.67	11.7	11.5	1.29–66.77
PM_2.5–10_ (μg/m^3^)	106 (14)	10.04 ± 4.07	9.9	5.9	1.76–22.38
Indoor PM mass					
PM_0.25_ (μg/m^3^)	104 (16)	10.45 ± 6.77	9.5	4.5	1.42–69.86
PM_0.25–2.5_ (μg/m^3^)	107 (13)	7.36 ± 4.57	6.5	5.7	0.77–30.86
PM_2.5–10_ (μg/m^3^)	111 (9)	4.12 ± 4.76	2.8	3.5	0.12–37.63

Abbreviations: IQR, interquartile range; Max, maximum; Min, minimum.
